# Does Cleaner Household Energy Promote Agricultural Green Production? Evidence from China

**DOI:** 10.3390/ijerph191610197

**Published:** 2022-08-17

**Authors:** Fanghua Li, Wei Liang, Dungang Zang, Abbas Ali Chandio, Yinying Duan

**Affiliations:** 1 College of Economics, Sichuan Agricultural University, Chengdu 611130, China; 2 School of Business & Tourism, Sichuan Agricultural University, Chengdu 611830, China

**Keywords:** cleaner household energy, agricultural green production awareness, agricultural green production level, Sichuan Province, iv-o-probit model

## Abstract

Cleaner household energy for agricultural green production can significantly alleviate energy poverty and food security, thus contributing to global sustainable development. Using survey micro-data collected from Sichuan Province, the ordered probit model, OLS model, and instrumental variables approach were applied for empirical analysis. The results show that: (1) cleaner household energy significantly enhances farmer’s agricultural green production awareness and improves agricultural green production levels, which is still significant after treating endogenous issues with the conditional mixing process estimation method and 2SLS model; (2) health plays a partially mediating effect of cleaner household energy on agricultural green production awareness and agricultural green production levels; (3) environmental protection awareness and digital literacy have a moderating effect and reinforce the positive impact of cleaner household energy on agricultural green production awareness and agricultural green production levels. This research suggests that governments can enhance the impact of cleaner household energy on agricultural green production through price and subsidy mechanisms.

## 1. Introduction

Household energy consumption and agricultural production are the two main sources of carbon emissions [1,2]. The long-term use of non-clean energy by households and traditional agricultural production practices produces large amounts of greenhouse gases, which are a major cause of frequent climate extremes [3]. In order to solve the issues of air pollution, environmental damage, energy poverty, health crises, and food security caused by non-clean energy consumption and traditional agricultural production, many countries are embarking on new “green revolutions” in the energy and agriculture sectors: cleaner household energy (CHE) [4] and agricultural green production (AGP) [5]. CHE can eradicate energy poverty [6], reduce harmful and greenhouse gas (GHG) emissions [7], accelerate human and social capital accumulation [8], and improve the health and well-being of the population [9]. AGP can also increase farmers’ income and alleviate poverty [10], reduce CO_2_ and environmental pollution [11], and improve the quality of agricultural production and food security indices [12]. Therefore, CHE and AGP are important ways to achieve the UN’s sustainable development goals (SDGs) of “no poverty”, “zero hunger”, “affordable and clean energy”, and “climate action” [13,14].

The existing literature on factors influencing farmers’ AGP has highlighted that the spread of digital technology [15] and the use of the Internet [16] has significantly increased the farmers’ awareness of AGP. Moreover, political identity [17], social networks [18], and social contacts [19] accelerate the accumulation of farmers’ social capital, which is positively correlated with farmers’ willingness to participate in AGP [18]. On the other hand, the adoption of modern agricultural-technology-related training programs can effectively help farmers to upgrade their production techniques and improve production ideas, increase human capital, and enhance the efficiency of AGP [20]. Non-farming work will lead to a reduction in the scale of family farming and breeding and farmers will prefer traditional production methods to green production [21]. Huang and Luo [22] argued that as income increases, households can afford greater fertilizer costs and thus increase fertilizer use, while Li et al. [19] found that increased farmer income incentivizes green agricultural production behavior. Based on panel data for 31 provinces in China from 1997 to 2017, Cui et al. [23] found that mechanization did not promote the greening of agriculture, whereas He et al. [24] conducted a study from the perspective of technological progress and concluded that the level of mechanization was positively related to AGP. In another study, Li [25] claimed that rural formal financing (bank credit) increases the agricultural green total factor productivity by improving the technological structure of agriculture. In contrast, Zhang et al. [26] revealed that credit has no significant effect on AGP. Using factor analysis and an ordered logistic model, Shi et al. [27] found that purchasing agricultural insurance significantly increased farmers’ willingness and intensity to participate in AGP.

There are some studies that discussed the effects of CHE on AGP. The long-term use of non-clean energy by households produces large amounts of CO_2_, triggering climate extremes that have a negative impacts on crop yields [28], forcing farmers to stop deforestation; protect the soil, vegetation, and water sources; and restore sustainable agricultural production [29]. Accelerating clean energy supply is one of the vital factors for green agriculture, hence CHE promotes AGP [30]. CHE can increase agricultural producers’ awareness of environmental protection, which in turn can lead to a reduction in the use of fertilizers, pesticides, and mulches in agricultural production [31], and an increase in the willingness to participate in AGP. Controlling agricultural surface pollution is an important part of AGP, and the use of biogas at home can reduce agricultural pollution [32]. An analysis of data from East Africa shows that the use of biogas not only reduces households’ use of fuel wood, thereby reducing deforestation, maintaining soil fertility, and increasing crop yields, but also reduces indoor air pollution and human damage, saving human capital and time costs for agricultural production [33]. A case study from Pakistan concluded that biological manure retains nutrients after digester fermentation and that the use of biogas can contribute to the reduction of commercial fertilizers and promote AGP [34]. Data from the 28 EU member countries for 2018 show a preference for liquid clean energy and a reduction in unexpected output (carbon) from the agricultural sector as clean energy use increases [35]. Wu et al. [36] conducted a seven-year follow-up experiment in an agricultural village called “Jiang jia Zhuang” in Northern China, the results of which have shown that the use of biogas at home promotes circular agriculture and reduces energy inputs and carbon emissions in agricultural production.

China is one of the world’s largest consumers of energy, and the consumption of non-clean energy sources has caused great damage to the ecological environment and hindered the country’s sustainable development. As a result, in September 2020, the Chinese government proposed a “Carbon Peak” goal for 2030 and a “Carbon Neutral” goal for 2060 (Double Carbon Goal), with the aims of reducing carbon consumption and emissions in economic development. The development of green agriculture is an important way to achieve the double carbon goal [37]. Provincial panel data statistics from 1997 to 2018 show that the level of green agriculture development in China shows a fluctuating upward trend [23,38], but there is significant regional heterogeneity, i.e., the highest in the east, followed by the west, and the lowest is in the center of China [39]. Located in Western China, Sichuan is one of the provinces with the highest level of household energy consumption and one of the largest regions for agricultural production in China. Currently, the level of green agricultural development in Sichuan has grown steadily and agricultural production has gradually transformed from traditional to modern [40]. Agricultural production in Central and Eastern Sichuan is dominated by the cultivation of grain crops (i.e., rice, maize, soybeans, and wheat) and pig breeding, making it an important grain-producing and pork-supplying area in China. Livestock farming has long been developed in Western Sichuan, making it an important region for the production of pastoral products in China. Sichuan Province has always been a typical “big agriculture” (agriculture, pastoralism, forestry, and fishing) region in China [41]. Therefore, this paper is representative of the study of the impact of CHE on AGP, using Sichuan as an example.

Although there are some studies discussing the effects of CHE on AGP, further refinement is needed. China is one of the world’s leading energy consumers and agricultural producers, but there is a relative lack of evidence for China in current studies on CHE and AGP. At the same time, most of the existing research on green agricultural development is based on a sample of macro statistics and there is a comparative lack of analysis of micro-data [42]. Therefore, the researchers took the survey micro-data from Sichuan Province as its sample and empirically analyzed whether and how CHE influences farmers’ AG which is rich in theoretical value. 

The remainder of this paper includes: an overview analysis of energy consumption and green agriculture development in Sichuan (Section 2); data and methods (Section 3); empirical analysis and discussion (Section 4); further research (Section 5); and conclusions and policy recommendations (Section 6).

## 2. An Overview of Energy Consumption and Green Agriculture Development in Sichuan Province

**Figure 1:**
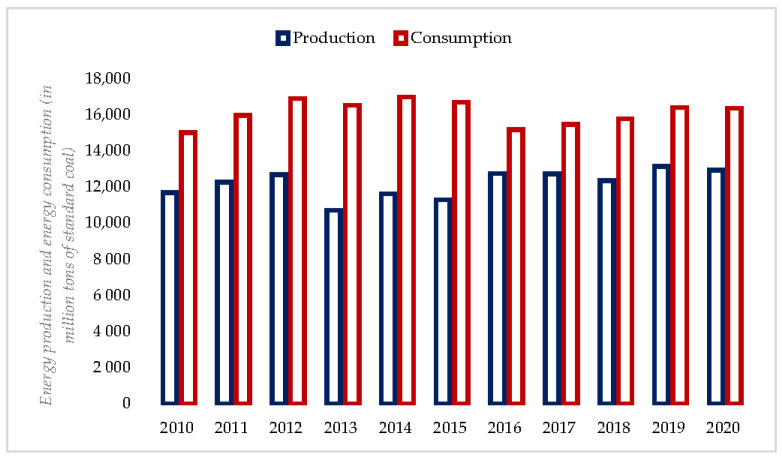
EP and EC statistics in Sichuan Province (2010–2020). **Source:** Sichuan Provincial Statistical Yearbook (2011–2021).

According to Figure 1, the changes in non-clean energy production (EP) and consumption (EC) in Sichuan Province from 2010 to 2020 are less volatile, maintaining an overall growth trend, and are consistently greater than the amount of energy produced, indicating that Sichuan Province consumes more energy and that non-clean energy still plays an important role in economic development.

**Figure 2:**
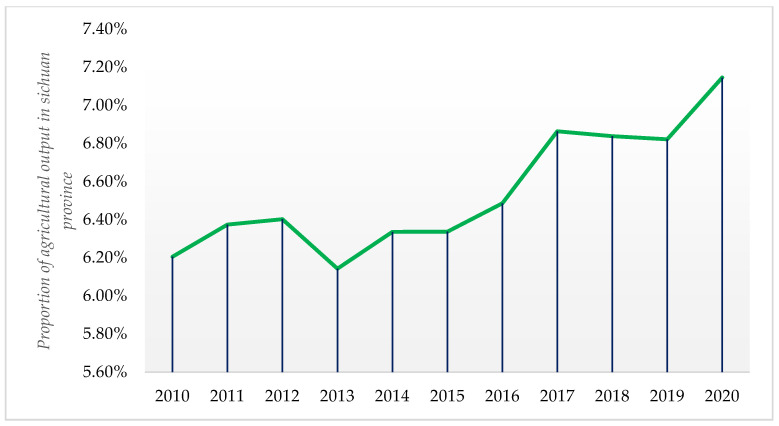
Proportion of agricultural output in Sichuan Province in China (2010–2020). **Source:** Sichuan Provincial Statistical Yearbook (2011–2021), China Statistical Yearbook (2011–2020).

According to Figure 2, the proportion of Sichuan’s agricultural GDP in the national agricultural GDP from 2010 to 2020 has generally maintained a growing trend and has increased significantly, illustrating the important role of Sichuan Province in China’s agricultural development.

**Figure 3:**
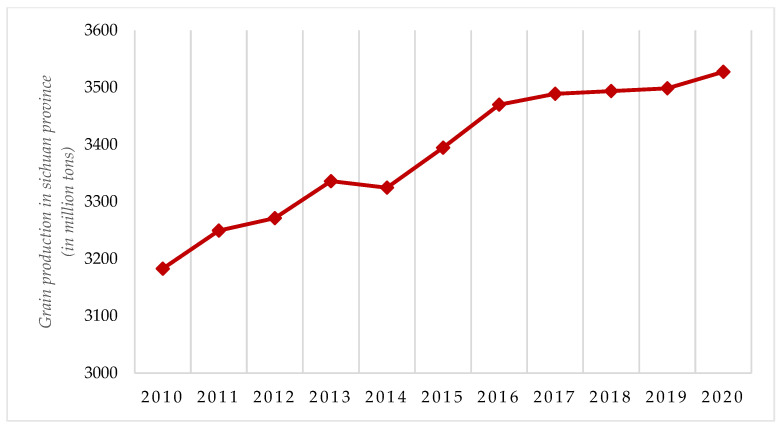
Grain production in Sichuan Province (2010–2020). **Source:** Sichuan Provincial Statistical Yearbook (2011–2021).

Figure 3 portrays the changes in grain production in Sichuan Province from 2010 to 2020. It can be seen that grain production in Sichuan Province is growing faster. The results in Figure 2 and Figure 3 show that the level of agricultural development in Sichuan Province has been increasing in recent years and significantly improving China’s agricultural development.

**Figure 4:**
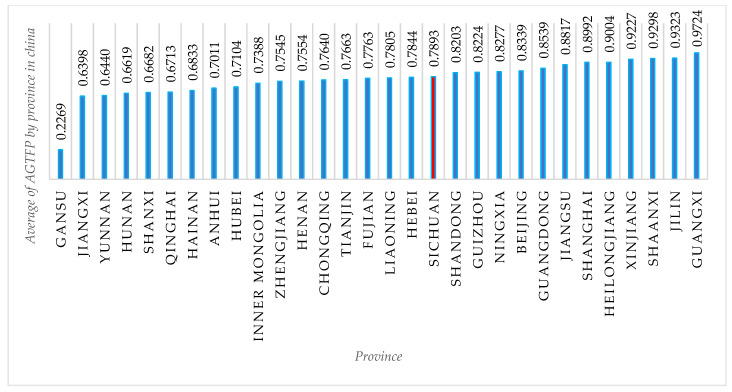
Average of AGTFP by province in China (2010–2019). **Source:** China Statistical Yearbook by Province (2011–2020).

Agricultural green total factor productivity (AGTFP) is a commonly used indicator at the macro level to measure the level of development of green agriculture, which covers the use of fertilizers, pesticides, and machinery in agricultural production, as well as carbon emissions [43]. This study used a static SBM model to calculate the AGTFP for each province in China from 2010 to 2019, and then calculated the ten-year average value of AGTFP for each province. Figure 4 portrays the ten-year AGTFP for 30 Chinese provinces (excluding Tibet, Taiwan, Hong Kong, and Macau), and it can be seen that the best development of green agriculture is in Guangxi province while the worst is in Gansu province, with Sichuan Province ranking 13th, indicating that green agriculture in Sichuan Province is at an intermediate level in China [40].

**Figure 5:**
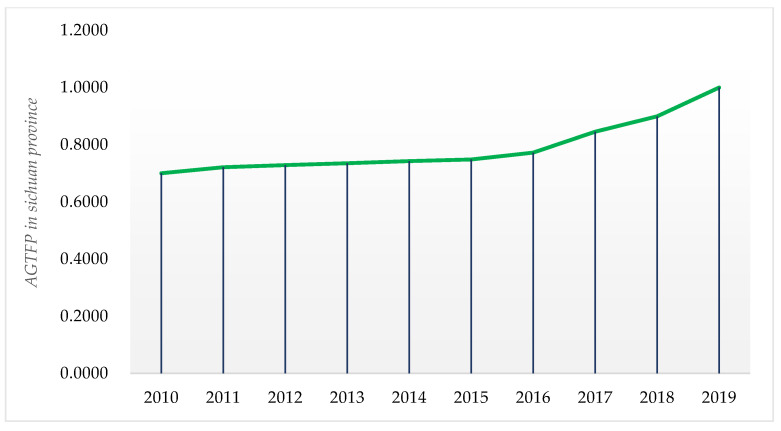
AGTFP in Sichuan Province (2010–2019). **Note:** AGTFP calculated by the static SBM model. **Source:** Sichuan Provincial Statistical Yearbook (2011–2020).

According to Figure 5, the AGTFP in Sichuan Province maintained a year-on-year upward trend from 2010 to 2019, indicating that the level of green agriculture development in Sichuan Province has improved faster and is at a higher level after 2017, which implies that agricultural production in Sichuan Province is gradually changing from traditional to green agriculture.

In summary, the macro data analysis shows that non-clean energy consumption and the level of green agricultural development in Sichuan Province are on the same growth trend. Does this mean that non-clean energy consumption can promote green agricultural development? Or, does cleaner energy consumption suppress AGP? This study addresses these questions using micro-data.

## 3. Data and Methods

### 3.1. Data

**Figure 6:**
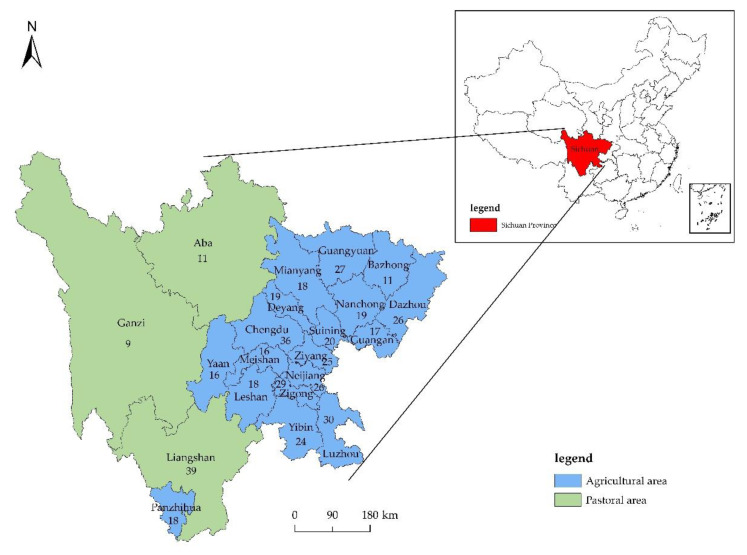
Study area and sample distribution. Note: The numbers in the figure, e.g., “Chengdu 36”, indicate that there are 36 sample data points from Chengdu in the empirical analysis of this work.

For its sample, this study uses data from the “Household Energy Consumption and Green Agriculture Development Survey” conducted in 21 cities (states) in Sichuan Province from January to April 2022. The survey was commissioned from the municipal (state) agricultural and rural bureaus and the township government departments and collected data were based on the basic personal and household information, agricultural (pastoral) production, health, and household energy use of farmers in the agricultural and pastoral areas of Sichuan Province. The survey actually distributed 600 questionnaires; 523 questionnaires were returned and 498 questionnaires were valid, accounting for 95.22% of the valid questionnaires. The reliability test value of the survey data of *Cronbach-α = 0.801*, and the validity test value of *KMO = 0.776* demonstrated that the survey data is reliable. First, according to the questions “Which of the following crops do you grow?” and “Which of the following livestock (poultry) do you raise?” if respondents selected both “not farming” and “not breeding”, respectively, they were not involved in agricultural production and were excluded from the data. Then, in the remaining sample, we refer to agriculture and pastoralism collectively as agricultural production. Later, we removed extreme values from the data, filled in missing values, and normalized the data. Finally, a total of 454 effective sample data points were obtained for empirical analysis. Figure 6 displays the study area and sample distribution of this research.

### 3.2. Variables

#### 3.2.1. Explained Variables: Agricultural Green Production (AGP)

In the current study, AGP is usually measured by indicators such as fertilizer [11], pesticide [44], green manure use [42], and straw treatment [45]. The common variables of AGP include “green production awareness” [18], “green production efficiency” [24], “green production willingness” [46], and “green production behavior” [47]. In this work, the researchers measured AGP in terms of “agricultural green production awareness (AGPA)” and “agricultural green production levels (AGPLs)”. AGPA refers to farmers’ knowledge of green production; the AGPL refers to farmers’ green production index. The specific definitions of explained variables are reported in Table 1.

#### 3.2.2. Explanatory Variables: Cleaner Household Energy (CHE)

Referring to Zang et al. [48], the researchers measures CHE in two ways in this work: First, compare the frequency of use of clean energy (FCE) with that of non-clean energy (FNCE), set “CHE_1 = 2” if FCE > FNCE, set “CHE_1 = 1” if FCE = FNCE, and set “CHE_1 = 0” if FCE < FNCE. Second, compare the proportions of clean energy (PCE) and non-clean energy (PNCE) in the household energy portfolio to consider a shift towards cleaner household energy if PCE > PNCE. The specific definitions of CHE are reported in Table 1.

#### 3.2.3. Control Variables

In this paper, personal characteristic variables such as “age” and “gender”, and household characteristic variables such as “number of agricultural labors (AL)” were se-elected as control variables. The specific definitions of all control variables are reported in Table 1.

The mean, standard deviation, and maximum and minimum values of all variables selected in this research are reported in Table 2. A total of 262 (57.71%) respondents have a high degree of agricultural green production awareness and 18.94% of farmers still lack agricultural green production awareness. The mean of the agricultural green production level is 0.52, the minimum value is −1.12, and the maximum value is 0.70, indicating that the overall agricultural green production level is low and the gap is obvious. More than half (50.88%) of households have a higher frequency of clean energy consumption than non-clean energy and 82.82% of households use a higher proportion of clean energy than non-clean energy, indicating that household energy consumption is shifting towards green. Most of the respondents are married (84.14%), middle-aged (average age was 42.33 years), male (72.25%), and have a lower education level. Only 97 (21.37%) have a high school or university education but 32.82% of family members have a college education. The mean duration that respondents have participated in agricultural production is about 22 years. Of those respondents, 82.16% have accepted agricultural production training and 296 (65.20%) have the experience of going outside to work. The mean land area of the interviewed households is 5.75 acres and 309 households (68.16%) have two laborers engaged in agricultural production. There is a large gap in the income of family agricultural production subsidies and most households (63.66%) do not purchase agricultural insurance. In the past 3 years, 15.42% of the households have suffered from agricultural disease and the agricultural production of 31.50% of the households has been negatively affected by COVID-19. The non-agricultural income of the interviewed households is relatively high and the gap is small (standard deviation = 0.60). More than half of the respondents (56.83%) were relatively healthy but 43.17% of the respondents have a low level of health, and 166 households (36.56%) have patients with respiratory illness. A total of 67.40% of the respondents have environmental protection awareness but the majority (64.10%) of the respondents have poor digital literacy.

### 3.3. Methods

The explained variable AGPA is an ordered multi-categorical variable and it is a commonly used econometric model, such as the ordered probit model (O-probit) [49]. The explained variable AGPL is the continuous variable and the classical model is ordinary least squares (OLS). So, we construct O-probit models for CHE_1 and AGPA (Equations (1) and (2)) and OLS models for CHE_1 and AGPLs (Equation (3)).

(1) $$AGPA_{i}^{*}=\omega_{1}+\beta_{1}\times CHE\_1+\lambda_{1}\times CV+\mu_{1}$$

(2) $$AGPA^{*}=\left\{\begin{array}{c} 1\;\;\;\;\;if\;0<i\leq 1\\ 2\;\;\;\;\;if\;1<i\leq 2\\ 3\;\;\;\;\;if\;2<i\leq 3\\ 4\;\;\;\;if\;\;3<i\leq 4\\ 5\;\;\;\;if\;4<i\leq 5 \end{array}\right.$$

(3) $$AGPL=\omega_{3}+\beta_{3}\times CHE_{1}+\lambda_{3}\times CV+\mu_{3}$$

We take AGPA as an example, referring to Wen et al. [50], and then construct the mediating effect and moderating effect model. Equations (4) and (5) represent the mediating role and moderating effect of some variables on the impact of CHE_1 on AGPA and AGPLs.

(4) $$\left\{\begin{array}{c} AGPA=\omega_{1}+\beta_{1}\times CHE\_1+\lambda_{1}\times CV+\mu_{1}\\ MV=\omega_{4}+\beta_{4}\times CHE\_1+\lambda_{4}\times CV+\mu_{4}\\ AGPA=\omega_{5}+\beta_{5}\times CHE\_1+\beta_{6}\times MV+\lambda_{5}\times CV+\mu_{5} \end{array}\right.$$

(5) $$\left\{\begin{array}{c} AGPA=\omega_{6}+\beta_{6}\times CHE\_1+\lambda_{6}\times CV+\mu_{6}\\ AGPA=\omega_{7}+\beta_{7}\times RV+\lambda_{7}\times CV+\mu_{7}\\ AGPA=\omega_{8}+\beta_{8}\times \left(CHE\_1\times RV\right)+\lambda_{8}\times CV+\mu_{8} \end{array}\right.$$

AGPA is agricultural green production awareness, AGPL is the agricultural green production level, CHE_1 is cleaner household production, MV is the mediating variables, RV is moderating variable, CV is the control variable, and *ω*, *β*, *λ* are regression coefficients.

## 4. Empirical Analysis

### 4.1. Basic Regression

In Table 3, the results of models (1) and (2) show that CHE significantly positively improved farmers’ AGPA, and the use of clean energy at home fosters farmers’ awareness and knowledge of AGP through the development of a green production mindset. The results for the control variables in model (1) show that REL significantly positive affected AGPA, implying that the higher the level of education, the greater the cognizance of AGP [18]. APT significantly positive influenced AGPA at the 1% level and the participation in agricultural knowledge and skills training can broaden farmers’ access to information related to AGP [51]. EWO significantly positive enhanced AGPA, and working outside the home broadens farmers’ minds to accept new things, making them capable of being more proactive in understanding AGP. AI is positively associated with AGPA at the 5% level, and the purchase of agricultural insurance means that farmers are already endowed with modern agricultural production characteristics, and this group of farmers is more receptive to AGP [27]. AD significantly reduces the AGPA of farmers, who may choose to increase pesticides or fertilizers use rather than diminish agricultural surface pollution when encountering pests and diseases. NAPI was significantly and negatively associated with AGPA at the 1% level; households with a higher NAPI have a lower willingness to participate in agricultural production [20] and do not spend time understanding AGP.

The results of model (3) in Table 3 show that CHE significantly increases farmers’ AGPL and clean energy household use saves household members’ time to participate in AGP, thus increasing AGPLs. The results of model (3), controlling for variables, further demonstrate that the lower odds of participating in AGP with farmers get higher, consequently decreasing the AGPL [22]. In contrast, APT upgrades farmers’ skills in agricultural production, increases their motivation to participate in AGP, and improves AGPLs. Likewise, EWO and AGPLs are significantly positively connected, and working outside the home provides access to more income and market information, increasing the subjective ability and willingness of farmers to participate in AGP [52]. AS significantly enhances AGPLs, and AS not only increases the capital element of AGP but also increases agricultural hedging of agricultural risk and increases AGP willingness [26]. In addition, AD and COVID-19 increase the difficulty and uncertainty of household production decisions, hence AD and COVID-19 significantly reduce AGPLs [53,54]. Finally, NAPI can increase AGPLs by alleviating the financial constraints on household participation in AGP.

### 4.2. Endogenous Treatment

The regressions of CHE_1 on AGPA and AGPLs may have endogenous issues due to omitted variables, reciprocal causality, etc., which may affect the results of the analysis. The instrumental variables method is the classic method for dealing with the endogenous problem in cross-sectional data. Therefore, the question “Has anyone in your household been diagnosed with a respiratory illness? (respiratory illness = RI) 1 = Yes, 0 = No” was selected as the instrumental variable (IV), and both Iv-O-probit and 2SLS models were employed to deal with potential endogenous problems. RI was chosen as IV because the household member with an RI would use less non-clean energy, thus promoting CHE but would not have an impact on the household’s agricultural practices, which means that RI is directly related to CHE but not to AGP, satisfying the exogeneity requirement of IV.

#### 4.2.1. Endogenous Issue Treatment of CHE and AGPA Regression

Currently, there are technical difficulties in directly using Iv-O-probit models to deal with the endogenous issue [55], and the conditional mixed process estimation (CMP) method is often applied to overcome technical constraints [56]. The results of the endogenous treatment of CHE_1 and AGPA are reported in Table 4. The value of the auxiliary estimation parameter atanhrho_12 is significantly different from zero (*p*-value = 0.00), indicating that the basic regression model has endogenous problems and that CHE_1 is an endogenous variable. The results of the first stage of Table 4 show that RI is significantly interrelated with CHE_1 and not with AGPA, satisfying the conditions of the IV method, and the F-value is 55.32, which is greater than the empirical value of 10, indicating that there is no weak IV problem. The second stage results show that CHE_1 still significantly enhances AGPA after dealing with the endogenous problem, with reduced coefficient values and average marginal effects at each cut-off point, indicating that the basic regression overestimates the effect of CHE_1 on AGPA.

#### 4.2.2. Endogenous Issue Treatment of CHE_1 Impact on the AGPL

The Hausman test for regression of CHE_1 on AGPLs was 0.035, which is less than 0.05, indicating that there is an endogenous problem in the basic regression, and CHE_1 is an endogenous variable. The results of the first stage of Table 5 illustrate that RI is significantly linked with CHE_1 but not with AGPLs, satisfying the IV method; the F-value is 103.2, which is greater than the empirical value of 10, indicating that there is no weak IV problem. The results of the second stage show that CHE_1 is still significantly positively associated with AGPLs after dealing with the endogenous issue, but the coefficients of AGPLs are reduced, implying that the effect of CHE_1 on AGPLs is overestimated in the basic regression.

### 4.3. Robustness Test

In this work, the researchers redefined CHE, i.e., replacing CHE_1 with CHE_2, and then conducted a robustness test regression of CHE_2 on AGPA and AGPLs. The results in Table 6 show that after replacing the core explanatory variables, CHE still significantly enhances AGPA, and significantly improves AGPLs. The results of the robustness tests for the control variables are essentially the same as in the basic regression and are not reported.

## 5. Further Research

### 5.1. Mediating Effect Analysis

CHE is very valuable to improving the health of household members [57], then reducing households’ expenditure on health care and increasing capital and labor factor inputs to the AGP [34], thereby having a positive effect on the AGP. Thus, health may play a mediating role in the effects of CHE_1 on AGPA and AGPLs. The researchers selected the data measurement “Health” with the question “Do you think you are currently healthy? a = 1 = very unhealthy, b = 2 = unhealthy, c = 3 = average, d = 4 = healthy, e = 5 = healthy”, and then takes “Health” as a mediating variable to conduct a mediating effect test. The results of model (3) in Table 7 show that CHE_1 is significantly and positively associated with “Health”, indicating that CHE improves the health of families. In models (4) and (5), both CHE_1 and “Health” were significantly and positively correlated with AGPA and AGPLs, which means that “Health” plays a partial mediating role in the effect of CHE_1 on AGPA and AGPLs. This result passed both the Sobel test and the bootstrap test.

### 5.2. Moderating Effect Analysis

Environmental protection awareness (EPA) is positively correlated with clean energy use and green production [22,31], implying that EPA may influence the effect of CHE on AGP. We chose the question “How does your household dispose of the waste generated in your life? a = dumping, b = burning, c = burying, d = disposal by a professional organization” to measure EPA; if “a”, “b”, and “c” are selected, the respondent is considered to have no EPA and is set to “EPA = 0”, and if “d” is selected, the respondent is considered to have EPA and is set to “EPA = 1”. EPA was used as a moderating variable to test whether EPA play a moderating role in the effect of CHE_1 on AGPA and AGPLs. Table 8 reports the results of the moderating effects analysis for EPA.

The results in Table 8 show that EPA was significantly and positively associated with AGPA and AGPLs, demonstrating that EPA significantly enhances farmers’ AGPA, and significantly increases AGPLs. The interaction term between EPA and CHE_1 was significantly positively interconnected with AGPA and AGPLs at the 1% level, indicating that EPA plays a moderating role in the effect of CHE_1 on AGPA and AGPLs, and reinforces the positive effect of CHE_1 on AGPA and AGPLs.

Farmers with high digital literacy (DL) have access to more information on clean energy use and green production from the Internet, and the use of the Internet can promote CHE [58] and AGP [16], meaning that DL may play a moderating role in the impact of CHE on AGP. We selected the question “Can you participate in online shopping through your smartphone or computer? a = yes, b = no”; if “a” is selected, the respondent is considered to have DL and is set to “DL = 1”, and if “b” is selected, the respondent is set to “DL = 0”. DL was used as a moderating variable to test whether DL plays a moderating role in the effect of CHE_1 on AGPA and AGPLs. Table 9 reports the results of the moderating effects analysis for DL.

The results in Table 9 demonstrate that DL was significantly and positively associated with AGPA and AGPLs, showing that DL significantly enhances farmers’ AGPA, and significantly increases AGPLs. The interaction term between DL and CHE_1 was significantly positively interconnected with AGPA and AGPLs at the 1% level, suggesting that DL plays a moderating role in the effect of CHE_1 on AGPA and AGPLs, and reinforces the positive effect of CHE_1 on AGPA and AGPLs.

## 6. Conclusions

### 6.1. Discussion

The current research assumes the belief that capital factors (financial and social capital) [18,19,25] and technological endowment [15,20] are the main factors affecting agricultural green production. At the same time, some of the literature discusses the impact of household energy consumption on agricultural green production. It is found that household energy cleaning can enhance farmers’ environmental awareness [31], control agricultural source pollution [32], and improve climate conditions [29], thereby promoting agricultural green production. This work used micro-data from China as a sample to explore the relationship between cleaner household energy and agricultural green production. A core conclusion of this study is that cleaner household energy significantly enhanced agricultural green production awareness and significantly improved agricultural green production levels. This means that cleaner household energy has a promoting effect on agricultural green production, and the conclusions of this work are similar to those of Ma and Yue [32], Yasar et al. [34], Wu et al. [36], etc. The contributions of this paper include: (1) improving research on this topic while also providing new references for future research; (2) not only discussing whether cleaner household energy affects agricultural green production but it also analyzing how cleaner household energy influences agricultural green production through the mediating effect model and moderating effect model. However, there are limitations to this research. The sample data is drawn from one province in China, and the conclusions drawn may only be applicable to China or developing countries. Therefore, a further direction would be to compare and analyze data from developing and developed countries to draw conclusions with differentiation.

### 6.2. Conclusions

Cleaner household energy and agricultural green production are an imperative part of China’s “carbon peaking and carbon neutrality goals”, as an important way for developing countries to alleviate poverty, address food security, improve livelihoods, and act as an essential means of combating global warming and environmental degradation. The core finding of this study is that cleaner household energy can significantly contribute to agricultural green production, which, with reference to the “green revolution” in the energy and agriculture sectors, can also change energy consumption and agricultural production decisions in the household sector.

This research used 454 points of micro-survey data from Sichuan Province as the sample to analyze the effects and mechanisms of cleaner household energy on agricultural green production awareness and agricultural green production levels by using O-probit and OLS models. The empirical analysis found the following: First, cleaner household energy significantly enhanced agricultural green production awareness and significantly improved agricultural green production levels, which is still valid after addressing potential endogenous issues with the instrument variables method and conducting robustness tests. Second, health partially mediates the impact of cleaner household energy on agricultural green production awareness and agricultural green production levels. Third, environmental protection awareness and digital literacy have a moderating role in the impact of cleaner household energy on agricultural green production and reinforce the positive impact of cleaner household energy on agricultural green production awareness and agricultural green production levels.

### 6.3. Policy Recommendations

Based on the conclusions of this paper, some policy recommendations are proposed as follows:

First, accelerate the development and conversion of clean energy, establish a sustainable and inclusive clean energy market mechanism, improve the construction of rural energy infrastructure, increase households’ willingness to use clean energy through a subsidy mechanism, and promote the transformation of household energy into clean energy.

Second, improve the policy design for green agricultural development, provide more policy support to farmers involved in green agricultural production, increase farmers’ willingness to produce green through a price mechanism, and improve farmers’ green production capacity through regular agricultural training.

## Figures and Tables

**Table 1:** Variable selection and definition.

Variable’s Type	Sub-Variables	Define
Explained variables: Agricultural Green Production (AGP)	Agricultural green production awareness (AGPA)	How much do you know about agricultural green production? (B31) 1 = Completely unknown; 2 = Largely unknown; 3 = Know some; 4 = General known; 5 = Completely known.
	Agricultural green production level (AGPL)	According to “Has your household’s fertilizer(B09), pesticides(B11), mulches(B14) and, mechanical fuel(B23) use in agricultural production increased, decreased or remained more or less the same in the last 3 years? a = increased = −1, b = remained unchanged = 0, c = decreased = 1; What are the main methods of disposal of waste mulch in your household? (B13) a = buried in situ = −1, b = open burning = −1, c = harmless disposal = 1; What are the main methods in which your household handles straw in farming and livestock production? (B15) a = straw return to the field (land) = 1, b = open burning = −1, c = for domestic indoor fuel = −1, d = processed as livestock feed = 1, e = processing and cultivation of edible mushrooms = 1; Has the amount of biological manure (green fertilizer) used in your household increased, decreased or remained more or less the same in the last 3 years? (B18) a = increased = 1, b = remained unchanged = 0, c = decreased = −1”. Using a subjective weighting method, we selected data from 7 questions and set “1 = green production, 0 and −1 = non-green production”, then the green production index was calculated using principal component analysis and factor analysis models to measure the level of agricultural green production.
Explanatory variables: Cleaner Household Energy (CHE)	Frequency of clean energy use (CHE_1)	According to the questions “How often does your household use firewood/grass/straw (C14), coal (C15), gas (C16), cow dung (C17), biogas (C18), natural gas (C19), LPG (C20), electricity (C21) and solar energy (C22) in daily life (cooking/heating/bathing)? a = never use = 1, b = hardly ever use = 2, c = occasionally use = 3, d = often use = 4, e = daily use = 5”; Then, the frequency scores for “C14–C17” were summed and set as the frequency of non-clean energy(FNCE) use, and the frequency scores for “C18–C22” were summed and set as the frequency of clean energy(FCE) use; Finally, FNCE and FCE were compared and if FCE < FNCE, assign a value of “0”, if FCE = FNCE, assign a value of “1”, if FCE > FNCE, assign a value of “2”.
	Proportion of clean energy use (CHE_2)	According to the question (C13) “What is the main fuel used in your household for daily living (cooking/heating/bathing)? a = wood/grass/straw, b = coal, c = gas, d = cow dung (‘a–d’ is non-clean energy); e = biogas, f = natural gas, g = LPG, h = electricity, i = solar energy (‘e–i’ = clean energy)”. First, if an option is chosen, it is assigned a value of “1 “ and “0” if no option is selected; Then, the values of options “a–d” are summed as the proportion of non-clean energy (PNCE) and the values of options “e–i” are summed as the proportion of clean energy (PCE); Finally, compare the PCE with the PNCE, assigning a value of “0” if the PCE < PNCE, a value of “1” if the PCE = PNCE, and a value of “2”if PCE > PNCE.
Control variables (CV)	Age	How old are you? (Unit: years)
	Gender	1 = Man; 2 = Woman.
	Marriage	1 = Unmarried; 2 = Married; 3 = Divorced; 4 = Death of wife/husband.
	Respondents’ education level (REL)	Your level of education is: 1 = Illiterate; 2 = Primary school; 3 = Junior high school; 4 = High/vocational school; 5 = Undergraduate/polytechnic; 6 = Master/doctor.
	Participation in agricultural production time (PAPT)	How many years have you been involved in agricultural or pastoral production? (Unit: year).
	Agricultural production training (APT)	Do you participate in agricultural training activities? 1 = Yes; 0 = No.
	Experiences of work outside (EWO)	Have you worked outside the home in the last 3 years? 1 = Yes; 0 = No
	Highest level of education in the household (HLEH)	What is your household member’s highest level of education? 1 = Illiterate; 2 = Primary school; 3 = Junior high school; 4 = High/vocational school; 5 = Undergraduate/polytechnic; 6 = Master/doctor.
	Agricultural land size (ALS)	How many acres of agricultural or pastoral land does your household have? (Unit: acre)
	Number of agricultural laborers (AL)	How many people in your household are permanently involved in agricultural or pastoral production?
	Agricultural subsidy (AS)	How much was your household’s agricultural subsidy last year in approximately RMB? (Agricultural subsidy). *Ln* (agricultural subsidy).
	Agricultural insurance (AI)	Does your household buy agricultural insurance in the last 3 years? 1 = Yes; 0 = No.
	Agricultural disease (AD)	Has your household agricultural production been affected by natural disasters/pests and diseases in the last 3 years? 1 = Yes; 0 = No.
	Agricultural production loan (APL)	Has your household applied for and received a loan from a bank for agricultural production in the last 3 years? 1 = Yes; 0 = No.
	Affected by COVID-19 (COVID-19)	Has the COVID-19 affected your household agricultural production? 1 = Yes; 0 = No.
	Non-agricultural production income (NAPI)	What was your household’s total income last year in approximately RMB? (Total household income); What was your household’s agricultural production income last year in approximately RMB? (Agricultural production income). *Ln* (Total household income—Agricultural production income)
Instrumental variable (IV)	Respiratory illness (RI)	Has anyone in your household been diagnosed with a respiratory illness? 1 = Yes,0 = No
Mediating variable( MV)	Health	Do you think you are currently healthy? 1 = very unhealthy, 2 = unhealthy, 3 = average, 4 = healthy, 5 = healthy.
Moderating variable (RV)	Environmental protect awareness (EPA)	How does your household dispose of the waste generated in your life? a = dumping, b = burning, c = burying, d = disposal by a professional organization, if “a”, ”b” and “c” are selected, it is set to “EPA = 0” = have no environmental protect aware-ness, if “d” is selected, it is set to “EPA = 1” = have environmental protect aware-ness
	Digital literacy (DL)	Can you participate in online shopping through your smartphone or computer? a = yes = DL = 1, b = no = DL = 0

**Note:** B09, B11, B13, B14, B15, B18, B23, B31, C13, and C14–C22 refer to the question numbers in the questionnaire. CHE_2 is for robustness testing.

**Table 2:** Variables’ descriptive statistics.

Variable	Observations	Percentage	Mean	Std. Dev.	Min	Max
**AGPA**	454	100.00%	3.39	1.08	1	5
AGPA = 1	43	9.47%				
AGPA = 2	43	9.47%				
AGPA = 3	106	23.35%				
AGPA = 4	220	48.46%				
AGPA = 5	42	9.25%				
**AGPL**	454	100.00%	0	0.52	−1.12	0.70
**CHE_1**	454	100.00%	1.41	0.66	0	2
CHE_1 = 0	44	9.69%				
CHE_1 = 1	179	39.43%				
CHE_1 = 2	231	50.88%				
**CHE_2**	454	100.00%	1.73	0.63	0	2
CHE_2 = 0	45	9.91%				
CHE_2 = 1	33	7.27%				
CHE_2 = 2	376	82.82%				
**Age**	454	100.00%	42.33	8.6	18	68
**Gender**	454	100.00%	0.72	0.45	0	1
Gender = 0	126	27.75%				
Gender = 1	328	72.25%				
**Marriage**	454	100.00%	2.04	0.51	1	4
Marriage = 1	35	7.71%				
Marriage = 2	382	84.14%				
Marriage = 3	21	4.63%				
Marriage = 4	16	3.52%				
**REL**	454	100.00%	2.77	1	1	6
REL = 1	38	8.37%				
REL = 2	140	30.84%				
REL = 3	179	39.43%				
REL = 4	60	13.22%				
REL = 5	26	5.73%				
REL = 6	11	2.42%				
**PAPT**	454	100.00%	22.16	8.98	0	45
**APT**	454	100.00%	0.82	0.38	0	1
APT = 0	81	17.84%				
APT = 1	373	82.16%				
**EWO**	454	100.00%	0.65	0.48	0	1
EWO = 0	158	34.80%				
EWO = 1	296	65.20%				
**HLEH**	454	100.00%	4.20	0.82	1	6
HLEH = 1	11	2.42%				
HLEH = 2	16	3.52%				
HLEH = 3	68	14.98%				
HLEH = 4	210	46.26%				
HLEH = 5	130	28.63%				
HLEH = 6	19	4.19%				
**ALS**	454	100.00%	5.75	4.36	1	30
**AL**	454	100.00%	2.13	0.62	1	5
AL = 1	42	9.25%				
AL = 2	309	68.06%				
AL = 3	71	15.64%				
AL = 4	18	3.96%				
AL = 5	14	3.08%				
**AS**	454	100.00%	6.99	1.69	0	8.7
**AI**	454	100.00%	0.36	0.48	0	1
AI = 0	289	63.66%				
AI = 1	165	36.34%				
**AD**	454	100.00%	0.15	0.36	0	1
AD = 0	384	84.58%				
AD = 1	70	15.42%				
**COVID-19**	454	100.00%	0.32	0.47	0	1
COVID-19 = 0	311	68.50%				
COVID-19 = 1	143	31.50%				
**NAPI**	454	100.00%	11.56	0.60	9.90	14.22
**RI**	454	100%	0.367	0.48	0	1
RI = 0	288	63.44%				
RI = 1	166	36.56%				
**Health**	454	100.00%	4.02	0.56	1	5
Health = 1	39	8.59%				
Health = 2	99	21.81%				
Health = 3	58	12.78%				
Health = 4	220	48.46%				
Health = 5	38	8.37%				
**EPA**	454	100.00%	0.67	0.47	0	1
EPA = 0	148	32.60%				
EPA = 1	306	67.40%				
**DL**	454	100.00%	0.49	0.5	0	1
DL = 0	291	64.10%				
DL = 1	163	35.90%				

**Table 3:** Regression results of CHE_1 and AGPA and AGPLs.

	O-Probit (1)	Marginal Effect of CHE_1 on the Impact of AGPA (2)					OLS (3)
Variables	AGPA	AGPA = 1	AGPA = 2	Variables	AGPA	AGPA = 1	AGPA = 2
CHE_1	0.199 **	−0.020 **	−0.013 **	CHE_1	0.199 **	−0.020 **	−0.013 **
	(0.085)	(0.009)	(0.006)	(0.011)	(0.012)	(0.018)	(0.038)
Age	0.001						−0.019 ***
	(0.015)						(0.005)
Gender	−0.098						−0.065
	(0.123)						(0.053)
Marriage	−0.141						−0.050
	(0.118)						(0.053)
REL	0.247 ***						0.041
	(0.078)						(0.033)
PAPT	0.007						0.004
	(0.012)						(0.003)
APT	1.960 ***						0.139 **
	(0.176)						(0.059)
EWO	0.229 *						0.139 ***
	(0.127)						(0.052)
HLEH	0.006						0.018
	(0.07)						(0.028)
ALS	0.008						0.001
	(0.016)						(0.007)
AL	0.024						0.022
	(0.010)						(0.040)
AS	0.010						0.025 *
	(0.038)						(0.012)
AI	0.287 **						0.089
	(0.130)						(0.058)
AD	−0.369 **						−0.069 *
	(0.165)						(0.038)
COVID-19	−0.154						−0.096 *
	(0.121)						(0.053)
NAPI	−0.475 ***						0.095 **
	(0.106)						(0.040)
Constant							−0.547
							(0.486)
R-squared							0.139
Observations	454	454					454

**Note:** standard errors in parentheses, *** *p* < 0.01, ** *p* < 0.05, * *p* < 0.1. AGPA = agricultural green production awareness; AGPL = agricultural green production level; CHE_1 = frequency of clean energy use = cleaner household energy; REF = respondents’ education level; PAPT = participation in agricultural production time; APT = agricultural production training; EWO = experience of work outside; HLEH = highest level of education in the household; ALS = agricultural land size; AL = number of agricultural laborers; AS = agricultural subsidy; AI = agricultural insurance; AD = agricultural disease; COVID-19 = affected by COVID-19; NAPI = non-agricultural production income.

**Table 4:** Results of the endogenous issue treatment for CHE and AGPA regression: Iv-O-probit with CMP method.

	First Stage		Second Stage					
	OLS (1)	O-Probit (2)	Iv-O-Probit (3)	Marginal Effect of CHE_1 on the Impact of AGPA with CMP (4)				
Variables	CHE_1	AGPA	AGPA	AGPA = 1	AGPA = 2	AGPA = 3	AGPA = 4	AGPA = 5
CHE_1		0.199 **	0.165 ***	−0.022 **	−0.014 **	−0.027 **	0.027 **	0.028 **
		(0.085)	(0.283)	(0.010)	(0.005)	(0.011)	(0.012)	(0.012)
RI	0.202 **	0.040	0.064					
	(0.086)	(0.116)	(0.049)					
CV	Control	Control	Control	Control	Control	Control	Control	Control
atanhrho_12			0.000	0.000	0.000	0.000	0.000	0.000
F-value	55.32							
Observations	454	454	454	454				

**Note:** standard errors in parentheses, *** *p* < 0.01, ** *p* < 0.05. AGPA = agricultural green production awareness; CHE_1 = frequency of clean energy use = cleaner household energy; RI = IV = respiratory illnesses; CV = control variables.

**Table 5:** Results of endogenous issue treatment for CHE and AGPL regressions: 2SLS model.

	First Stage		Second Stage
	OLS (1)	OLS (2)	2SLS (3)
Variables	CHE_1	AGPL	AGPL
CHE_1		0.081 **	0.078 **
		(0.038)	(0.0311)
RI	0.202 **	−0.0287	−0.0287
	(0.0860)	(0.0514)	(0.0504)
CV	Control	Control	Control
Constant	0.044 **	−0.503	−0.523
	(0.0193)	(0.477)	(0.567)
F-value	103.2		
R-squared	0.159	0.140	
Observations	454	454	454

**Note:** standard errors in parentheses, ** *p* < 0.05. AGPL = agricultural green production level; CHE_1 = frequency of clean energy use = cleaner household energy; RI = IV = respiratory illnesses; CV = control variables.

**Table 6:** Robustness test results of CHE and AGPA and AGPL regressions.

	O-Probit (1)	Marginal Effect of CHE_2 on the Impact of AGPA (2)					OLS (3)
Variables	AGPA	AGPA = 1	AGPA = 2	AGPA = 3	AGPA = 4	AGPA = 5	AGPL
CHE_2	0.169 **	−0.026 **	−0.014 **	−0.016 **	0.043 **	0.030 **	0.130 ***
	(0.076)	(0.011)	(0.006)	(0.008)	(0.018)	(0.014)	(0.037)
CV	Control	Control	Control	Control	Control	Control	Control
R-squared							0.125
Observations	454	454					454

**Note:** standard errors in parentheses, *** *p* < 0.01, ** *p* < 0.05. AGPA = agricultural green production awareness; AGPL = agricultural green production level; CHE_2 = proportion of clean energy use = cleaner household energy; CV = control variables.

**Table 7:** Results of mediating effect tests for the effect of CHE_1 on AGPA and AGPLs: Health.

	Before Mediating Effect Treatment		MV_1	After Mediating Effect Treatment	
	O-Probit (1)	OLS (2)	O-Probit (3)	O-Probit (4)	OLS (5)
Variables	AGPA	AGPL	Health	AGPA	AGPL
CHE_1	0.199 **	0.081 **	0.187 **	0.171 **	0.128 ***
	(0.085)	(0.038)	(0.085)	(0.085)	(0.037)
Health				0.082 ***	0.044 **
				(0.029)	(0.021)
CV	Control	Control	Control	Control	Control
Constant		−0.547			−0.397 **
		(0.486)			(0.190)
R-squared		0.139			0.127
Sobel test (*p*)				0.022 < 0.05	0.037 < 0.05
Bootstrap (500)					
In-direct effect				0.075 **	0.013 **
				(0.035)	(0.006)
Direct effect				0.081 **	0.128 **
				(0.039)	(0.055)
Observations	454	454	454	454	454

**Note:** standard errors in parentheses, *** *p* < 0.01, ** *p* < 0.05. AGPA = agricultural green production awareness; AGPL = agricultural green production level; CHE_1 = frequency of clean energy use = cleaner household energy; MV_1 = mediating variable = health; CV = control variables.

**Table 8:** Results of moderating effect tests for the effect of CHE on AGPA and AGPLs: EPA.

	O-Probit (1)	O-Probit (2)	OLS (3)	OLS (4)
Variables	AGPA	AGPA	AGPL	AGPL
CHE_1	0.160 **	0.173 **	0.078 **	0.094 **
	(0.076)	(0.082)	(0.038)	(0.027)
EPA	0.240 **	0.196 **	0.196 ***	0.159 ***
	(0.108)	(0.091)	(0.053)	(0.043)
CHE_1 × EPA		0.208 ***		0.141 ***
		(0.059)		(0.025)
CV	Control	Control	Control	Control
Constant			−0.132 ***	−0.078 **
			(0.045)	(0.034)
R-squared			0.131	0.125
Observations	454	454	454	454

**Note:** standard errors in parentheses, *** *p* < 0.01, ** *p* < 0.05. AGPA = agricultural green production awareness; AGPL = agricultural green production level; CHE_1 = frequency of clean energy use = cleaner household energy; EPA = environmental protection awareness; CV = control variables.

**Table 9:** Results of moderating effect tests for the effect of CHE on AGPA and AGPLs: DL.

	O-Probit (1)	O-Probit (2)	OLS (3)	OLS (4)
Variables	AGPA	AGPA	AGPL	AGPL
CHE_1	0.186 **	0.224 **	0.120 ***	0.168 ***
	(0.077)	(0.103)	(0.038)	(0.050)
DL	0.317 ***	0.439 *	0.160 ***	0.363 **
	(0.102)	(0.239)	(0.048)	(0.143)
CHE_1×DL		0.212 ***		0.089 ***
		(0.062)		(0.025)
CV	Control	Control	Control	Control
Constant			−0.286 ***	−0.367 ***
			(0.072)	(0.090)
R-squared			0.136	0.199
Observations	454	454	454	454

**Note:** standard errors in parentheses, *** *p* < 0.01, ** *p* < 0.05, * *p* < 0.1. AGPA = agricultural green production awareness; AGPL = agricultural green production level; CHE_1 = frequency of clean energy use = cleaner household energy; DL = digital literacy; CV = control variables.

## Data Availability

The datasets used or analyzed during the current study are available from the corresponding author on reasonable request.
